# Influence of EDC on Dentin-Resin Shear Bond Strength and Demineralized Dentin Thermal Properties

**DOI:** 10.3390/ma9110920

**Published:** 2016-11-12

**Authors:** Lin Tang, Yi Zhang, Yuhua Liu, Yongsheng Zhou

**Affiliations:** Beijing Key Laboratory of Digital Stomatology, National Engineering Laboratory for Digital and Material Technology of Stomatology, Department of Prosthodontics, Peking University School and Hospital of Stomatology, Beijing 100081, China; pkutanglin91@163.com (L.T.); zydentist@163.com (Y.Z.); kqzhouysh@hsc.pku.edu.cn (Y.s.Z.)

**Keywords:** shear bond strength, carbodiimide, resin–dentin bond, differential scanning calorimetry

## Abstract

This study aimed to evaluate the bonding strength and thermal properties of demineralized dentin with and without EDC treatment. Sound human molars were randomly divided into seven treatment groups (*n* = 20): control, 80% ethanol, and five EDC ethanol solutions (0.01–1.0 M). In each group, 16 samples were used for bond strength assessment and 4 samples were used for scanning electron microscopy (SEM) analysis. A further 70 intact molars were used to obtain a fine demineralized dentin powder, treated with the same solutions and were evaluated the crosslink degree by ninhydrin test and denaturation temperature (*T*_d_) by differential scanning calorimetry. EDC-treated specimens (<1.0 M) had a higher bond strength, especially 0.3 and 0.5 M group, than the control counterpart. There was a significant drop in bond strength of 1.0 M EDC group. SEM revealed a homogeneous and regular interface under all treatments. EDC treatment significantly increased the demineralized dentin cross-link degree and *T*_d_ compared with the control and ethanol treatments. The 0.3 and 0.5 M treatments showed the highest cross-link degree and *T*_d_. In terms of mechnical and theramal properties consideration, 0.3 and 0.5 M EDC solutions may be favorable for when applied with etch-and-rinse adhesives, but it is still needed further long-term study.

## 1. Introduction

Dentin bonding degradation has long been challenging dentistry practitioners and is currently in the multidisciplinary spotlight. Researchers have posited many strategies for obtaining a more durable and steady bonding interface [1,2,3,4]. As we know, etch-and-rinse adhesive has long been thought as a gold standard in dental bonding, as it has a better performance than the self-etch system. Single Bond 2, as two-step total-etch bonding agent, is widely used in clinical work. Owing to the influence of the acid capability of 37% phosphoric acid (pH ≈ 0.4) used for etching in Single Bond 2 bonding techniques, imperfect infiltration of the hydrophilic resin monomers occurs, which may cause a defective zone at the bottom of the hybrid layer, with a number of collagen fibrils and matrix-bound MMPs being exposed [5,6,7]. Previous studies have proposed several innovative techniques for resin–dentin bonding, but no one method can achieve perfect mechanical properties and completely prohibit deterioration of the dentin/adhesive interface, and hence biodegradability still seems to be inevitable [8,9,10,11]. Recently, both physical and chemical cross-linking methods, have shown good performance in some in vitro studies [12,13,14,15,16,17,18,19].

Ultraviolet activation (UVA) of riboflavin has been proven effectively in cross linking collagen [14,18]. Whereas, the physical method does not gain its popularity in clinically relevant work with UV light harmful effects. Glutaraldehyde, as a classical synthetic cross-linker, its high cytotoxity has limited its clinical application routinely [20,21]. Carbodiimide, namely 1-ethyl-3-(3-dimethylaminopropyl) carbodiimide, as one of the most promising and commonly used artificial cross-linkers in bio-modification, has attracted much attention since it was introduced into bonding to dentin tissues in recent decades [22,23,24]. It has been used as an alternative cross-linking agent because of its good bio-compatibility [20]. However, the principle of EDC cross-linking role is: the RN=C=NR group reacts with ionized carboxyl groups in proteins to form an *O*-acylisourea intermediate that can react with a non-proteinated amino group and an adjacent protein chain to form a stable covalent amide bond between proteins [25,26]. It has been found that EDC requires a relatively long time (at least 1 h) to cross link, which may make it clinically impractical [27]. However, Mazzoni et al. observed that when EDC is applied with total-etch adhesive, even 1 min pre-treatment time preserves the dentin-resin bonds mechanical properties [22,23]. Furthermore, Tezvergil-Mutluay et al. found that 1 min of EDC coating can induce satisfactory cross-linking of collagen fibrils, as well as inhibit matrix metalloproteinases (MMPs) at neutral pH [5,28]. In our recent study, several EDC solutions (0.1, 0.3, 1.0 and 2.0 M) have been applied, and we find the dentin surface with ethanol-based EDC solution (≤1 M) before resin composite bonding may improve dentin-resin micro-tensile bond strength [29]. However, a clear protocol of EDC dentin surface coating is still questionable [22,23]. Little attention has been focused on investigating the influence of EDC on the dentin shear bond strength, one of the most essential, valuable and reliable characteristics for initial screening of new dental adhesive systems [30,31].

The dentin organic substrate is mainly composed of type I collagen, the integrity and stability of which is a vital factor in dentin bonding longevity. The collagen networks can be cross-linked by EDC, thus increasing the resistance to enzymatic or physicochemical degradation. To our knowledge, there are few studies focusing on the thermal ability of dentin organic components with EDC cross-linking treatment, one of the biological behaviors [16,24].

Thus, this study was conducted to evaluate the shear bond strength of demineralized dentin with Single Bond 2, the cross-link degree and the thermal stability of demineralized dentin with and without EDC treatment. The null hypothesis tested was that EDC pretreatment concentration has no effect on the dentin shear bond strength with the etch-and-rinse adhesive Single Bond 2 and the demineralized dentin cross-link degree and *T*_d_.

## 2. Results

### 2.1. Shear Bond Test

#### 2.1.1. Shear Bond Strength

**Figure 1:**
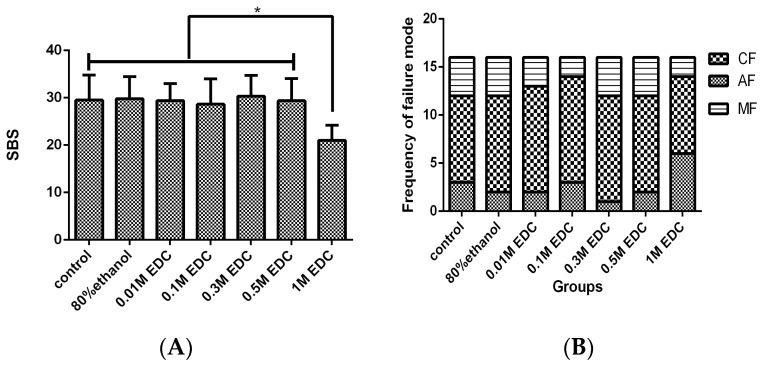
(**A**) The dentin shear bond strength with Single Bond 2 for different surface treatments. The asterisk indicates a significant difference in SBS between pretreatments (Tukey’s test, *p* < 0.001). The dentin bond strength of the 1.0 M EDC group was distinctly lower than the corresponding data that was obtained for the other surface treatment groups; (**B**) The failure mode frequency of the SBS test for different treatment groups, where no significant difference was found. * indicates significant difference compared with 1.0 M EDC group (*P* < 0.05).

The shear bond strength (SBS) results of the EDC pretreatment technique are summarized in Table 1 and Figure 1A. The mean SBS of all groups ranged from ~21 to 30 MPa. The SBS of the 0.01 to 0.5 M EDC treatment groups showed no significant change compared with the control and ethanol treatments. Among the treatment groups, the 0.3 M EDC group had the highest value, followed by the 0.5 M EDC group. The SBS was significantly influenced by the 1.0 M EDC concentration (*P* < 0.0001); specifically, the mean SBS dropped dramatically compared with the other groups.

#### 2.1.2. Mode of Failure

The failure mode distribution is given in Figure 1B. Most of the failure modes were adhesive failure. The chi-squared test of the failure mode frequency of the SBS test specimens indicated that the failure mode frequency was not influenced by the surface treatment method (*P* > 0.05).

### 2.2. SEM Analysis

**Figure 2:**
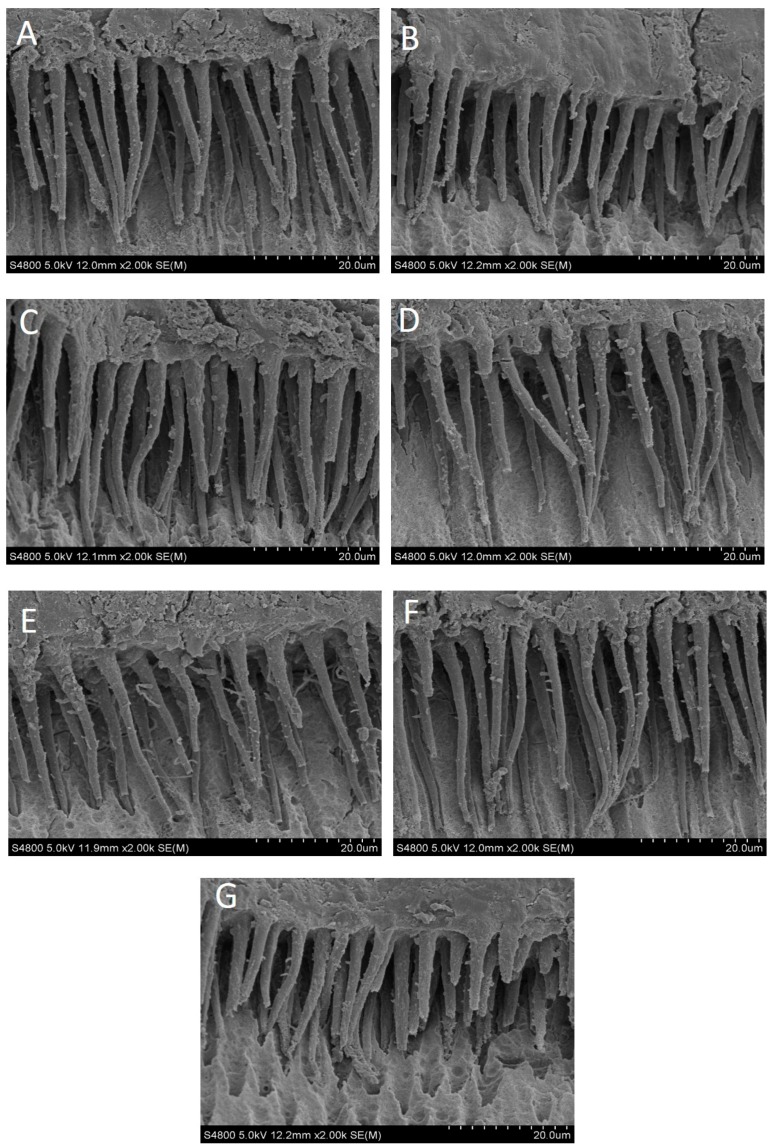
Representative SEM micrographs of the resin–dentin interface obtained after 24 h storage of specimens treated with different concentrations of ethanol-based EDC in 0.9% NaCl solution. Original magnification ×2000. (**A**) No treatment (control) group; (**B**) ethanol treatment group; (**C**) 0.01 M EDC treatment group; (**D**) 0.1 M EDC treatment group; (**E**) 0.3 M EDC treatment group; (**F**) 0.5 M EDC treatment group; (**G**) 1.0 M EDC treatment group.

As shown in Figure 2, intact and complete hybrid layers were found in the EDC treatment specimens regardless of the solution concentration. Some branches of dentine tubules were infiltrated by adhesive resin in each group. Shorter resin tags were seen within the resin-dentin joints in the ethanol and 1.0 M EDC treatment group specimens. In addition, some longer branches of dentine tubules were easily seen in 0.3 M group.

### 2.3. Cross-Link Degree Evaluation and Differential Scanning Calorimetry Analysis

**Figure 3:**
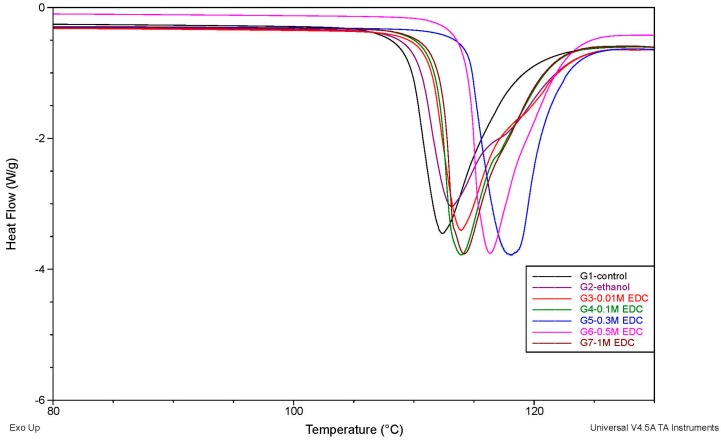
DSC thermograms of the demineralized dentin powder with and without EDC treatment. The first endothermal peak illustrates the thermal degradation temperature of the specimens, mainly composed of type I collagen.

Table 2 clearly shows that EDC-treated demineralized dentin powder had a significantly higher cross-link degree and *T*_d_ than the untreated and ethanol groups, while, no difference was found between the control and ethanol treatment groups. When the reagent solution concentration (<0.5 M) increased, the cross-link degree and *T*_d_ also increased, and the 0.3 M EDC-treated specimens showed the highest *T*_d_. However, a decline in *T*_d_ occurred at 1.0 M EDC concentration. Figure 3 shows the representative DSC thermograms of the test samples. In every sample DSC test, an endothermal peak was detectable, which was characterized as the *T*_d_ of type I collagen.

## 3. Discussion

With the development of adhesive restorative dentistry, many new dental bonding materials were produced. The etch-and-rinse approach is one of the most frequently employed in clinical work. In the present study, treatments with different EDC concentrations for 1 min, which may be realized clinically, were tested to explore the effect of EDC on the dentin shear bond strength with a classical etch-and-rinse adhesive system—Single Bond 2.

EDC is a water- and ethanol-soluble carbodiimide, usually obtained as the hydrochloride, that has widely been used in the cross-linking of collagen in biomedical materials, as well as a new EDC cross-linked collagen membrane for use in guided bone regeneration and guided tissue regeneration [32]. This so-called zero-length cross-linkage can directly and irreversibly change the three-dimensional structure of catalytic domains of MMPs and other endogenous enzymes, naturally lowering their enzymatic activity [22,23]. It has been shown in our recent study that ≤1.0 M EDC has a significant dentinal collagen cross-linking potential, and but favorable concentration needed to be tested further, as higher concentrations chosen for dentin treatment may have a toxic effect [23,25,29,33]. Scheffel et al. have also provided evidence that 0.1, 0.3, and 0.5 M EDC do not cause transdentinal cytotoxicity of MDPC-23 cells (odontoblast-like cells), showing that EDC has good biocompatibility [20,21]. To explore the effects of lower and higher concentrations of EDC on dentin surface treatment, both lower (0.01 M) and closer (0.1, 0.3 and 0.5 M) and higher (1.0 M EDC) concentrations were also investigated in the present study.

Specimens of micro-tensile method are easily dehydrated and damaged, with more pre-test failure [34,35], so shear bond test was applied in the study to evaluate the mechanical properties of immediate dentin-resin bonds. In general, so called micro-specimens are bonding areas below 1 mm^2^ [36] or 2 mm^2^ [34] or 3 mm^2^ [37]. The researchers tended to believe that the micro-shear bond strength was not better than the conventional bond strength, as there are many factors that cannot be controlled perfectly in the micro-SBS [30]. For example, with the difficulties in the micro-SBS specimen preparation, the adhesive layer tended to be thicker, the stress distribution was highly non-uniform, and the fracture was initialized by the tensile stress produced at load application [35]. Furthermore, it is common sense that a larger bonding area will have a greater possibility of adhesive layer flaws, where the load application would trigger the pathway of failure [34]. To balance the bonding homogeneity and the specimen representativeness, we designed the shear bond sample under quality control, as described in the Materials and methods, to reach *r* = 1 mm, as small as possible, and to simultaneously obtain the adhesive layer as thin and homogenous as possible.

Ekambaram et al. compared different solvents of EDC, suggesting that EDC in acetone could enhance its dentin collagen cross-linking potential, but they did not specify the concentration of ethanol solution in their study [38]. Ethanol was chosen as the solvent in present study because it has a higher vapor pressure (40 mmHg) than water (17 mmHg) at room temperature, which facilitates the removal of residual water. Otherwise, water phase or separation will most likely happen, and result in the loss of integrity of the bonding interface, further damaging the biomechanical properties in the short term and leading to bonding failure in the long term. Moist dentin surfaces can prevent collagen from collapsing, and ethanol is effective at replacing the surrounding water molecules in inter- or intra-collagen fibers. Another important consideration was dental material properties, because differences in the behavior of bonding systems may be related to their compositions, such as type of solvent. Dental adhesive systems consist of many different components. Single Bond 2 is a water-ethanol solvent system. The principle of EDC cross-linking capacity means that carboxyl groups should be ionized. The performance of carbodiimides in absolute ethanol would be different from that in water. It has been reported that the optimal ethanol concentration in the ethanol-water mixtures is around 80 *v*/*v*% [26]. To avoid an initial reaction between the bonding agent and acetone, and based on the two considerations mentioned above, we chose 80% ethanol as the EDC solvent. Furthermore, 80% ethanol is clinically feasible.

In the current study, apart from 1.0 M group, the SBS of the EDC treatment groups showed no significant difference. And it was further confirmed that low EDC concentrations (<1.0 M) have no adverse effect on the immediate dentin SBS with Single Bond 2. This offers us possibility to further explore the durability of the EDC role on the long-term dentin-resin bonds. In terms of the mechanical properties, previous studies have established an apparent drop in the bond strength in high concentration (>1.0 M) EDC treatments, which is consistent with our finding in 1.0 M group [20,21,29]. Scheffel et al. reported that the cross-link role of EDC is concentration dependent in their work on different concentrations of EDC in vitro to inhibit the activity of MMPs [5]. This may be explained by the fact that a higher concentration may lead to more molecular cross-linkage. In lieu of our current knowledge, the adverse effect of higher concentration EDC treatment on mechanical properties could be explained by the limited amount of carboxyl groups and/or the presence of molecular reaction sites on the dentin matrix for interaction with or linked by the EDC. As the ethanol solvent solution evaporates, EDC molecules can crystallize more easily (surface deposition of EDC molecules) in the higher concentration EDC group, thus in turn critically impairs the infiltration of the adhesive into the treated dentinal tissue and inter-fibril space. It is widely accepted that ethanol wet bonding can drastically improve the tensile bond strength [39]. However, in our investigation, this phenomenon did not occur. A possible rationale is that the ethanol application protocol was not in accordance with classical ethanol wet bonding technique [40].

Failure mode observation and SEM analysis were further conducted to identify what the mechanical properties substantiated. The present data showed no exact correlation among the treatment and failure modes, distribution. De-bonding interface observation results showed more adhesive fracture, followed mixed fracture and cohesive fracture. The SEM morphological observations revealed shorter resin tags and more adhesive fracture in the ethanol and 1.0 M EDC treatment. It is widely accepted that there is no correlation between the length of resin tags and bond strength [41,42]. This can be explained as that the SEM specimens evaluated surface was not so perpendicular to the bonding interface even if we only chose the 2–3 mm area above the pulp chamber to analyzed. However, it is important to mention that the EDC role did not affect the adhesive resin infiltration in the micro-view.

The integrity of the collagen network, and the thermal stability, with its predictability of clinical performance, is one of the crucial characteristics. Using sensitive differential scanning calorimetry (DSC), the thermal properties of dentin substrates, especially the thermal denaturation temperature (*T*_d_) of the dehydrated organic phase, can be characterized. *T*_d_ serves as an indirect indicator of the cross-linking role when the difference in the amount of heat required to increase the temperature of demineralized dentin materials is measured as a function of temperature. *T*_d_ is directly related to collagen cross-link degree which can be determined by ninhydrin test. The study hypothesis was rejected, as the EDC-treated specimens had a significantly higher cross-link degree and *T*_d_ (Table 2), and these findings were corroborated by a previous study [43,44]. The demineralized dentin powder, as the organic material, is mainly composed of type I collagen, and the remaining ground substance includes dentine-specific proteins. The increasing trend of cross-link degree and *T*_d_ of samples after the low cytotoxicity cross-linker EDC treatment clearly illustrates that the EDC treatment has an important role in protecting the structure of the demineralized organic constituents, and promotes the resistance of thermal degradation by increased covalent binding inter- and intra-collagen fibrils. These results were also consistent with a previous study by Cadenaro et al. [45].

It was interesting to notice that the increasing cross-link degree and *T*_d_ was also concentration dependent, and the drop of cross-link degree and *T*_d_ in the 1.0 M EDC treatment group could also boil down to the limited carboxyl and amino groups in the samples reaching the cross-linking reaction plateau [25]. It was worth mentioning that the cross-link degree of 1.0 M group was significantly lower (*P* < 0.05) than that of 0.3 and 0.5 M group, while the *T*_d_ was no difference among the three groups. The issue is explained as ninhydrin test is used to evaluate the amount of free amino groups in each group, and it would not be affect by the excess EDC molecules; while *T*_d_ analysis may be influenced by that. As illustrated above, the higher cross-link degree and *T*_d_ of 0.3 and 0.5 M, an direct and indirect index of collagen mechanical property, were evidence of dentin substrate stability and durability promotion in the long term.

This study was the first to use fine (15–30 μm) homogeneous dentin powder as the test samples in DSC analysis. In previous studies that used dentin disks as the specimens [43,44,45], the heterogeneity of the heat distribution at the crucible could result in cracks and micro-motion, and such uncontrollable external factors throughout the whole heating processing brought about more complex alterations in the thermograms. In contrast, the use of fine powder ultimately reduced these possibilities to a large extent.

Within the limitation of this present study and taking the shear bond test into account, overall, the results suggest that the 0.3 and 0.5 M EDC concentrations may be favorable for coating demineralized dentin when applied with Single Bond 2. But our finding only showed the initial effect in terms of both mechanical properties and collagen network structure stability. Long term effect of EDC treatment on dentin-resin bonding studies are needed to be done to further determine the favorable EDC characteristics for dentin surface modification.

## 4. Experimental Section

### 4.1. Materials

Single Bond 2 (3M ESPE, St. Paul, MN, USA. Lot: N463807) (two-step etch-and-rinse); Filtek Z250 (3M ESPE, St. Paul, MN, USA. Lot: 1370A3); 0.5 wt % aqueous solution of chloramine-T (Peking University School and Hospital of Stomatology, Beijing, China). EDC hydrochloride (Sino-pharm Chemical Reagent Co., Ltd., Beijing, China); 80% (*v*/*v*) ethanol solution (Peking University School and Hospital of Stomatology, Beijing, China); ninhydrin solution (Sino-pharm Chemical Reagent Co., Ltd., Beijing, China). EDC powder was dissolved and diluted with dehydrated 80% ethanol to prepare a series of solutions of increasing ethanol-based EDC concentrations (0.01, 0.1, 0.3, 0.5, and 1.0 M). All prepared EDC reagents were stored at 4 °C in a light-tight, light-resistant container until experiments for no more than 2 days.

### 4.2. Methods

#### 4.2.1. Tooth Preparations

Ethical approval (PKUSSIRB_201523093) for the study was obtained from the Biomedical Ethics Committee of the School and Hospital of Stomatology, Peking University. From patients ranging from 18 to 40 years old who had provided written informed consent in Oral and Maxillofacial Surgery, 190 extracted sound human molars were collected, of which 140 were used for shear bond strength and SEM analyses, and the other 50 were used for DSC analysis. All external debris was removed using a hand scaler, and the teeth were stored in a 0.5% aqueous solution of chloramine-T at 4 °C and utilized within 1 month of extraction.

#### 4.2.2. Shear Bond Test and SEM Analysis

Enamel removal of the 140 molars was carried out using a low-speed saw (Isomet 1000, Buehler Ltd., Lake Bluff, IL, USA) and under-water irrigation to expose the flat mid-coronal dentin surface. The surface of all teeth were polished with SiC paper (Panda, Beijing East New Grinding Tools Co., Ltd., Beijing, China), gradually from 120, 200, 400, to 600 grit each for 20 s, under flowing water to achieve a uniform mid-coronal dentin surface. Subsequently, all the specimens were randomly divided into seven groups (*n* = 20) according to the surface treatment: no pretreatment (control), 80% ethanol treatment, and five concentrations of EDC ethanol solutions.

For the shear bond test specimens, the dentin surfaces were acid etched with 37% phosphoric acid (pH = 0.4) for 15 s, rinsed thoroughly with deionized water for 10 s, and kept moist. Six groups of the demineralized dentin surfaces were treated with and without the different surface treatments: the five progressive concentrations (0.01, 0.1, 0.3, 0.5, and 1.0 M) of EDC ethanol solution and 80% ethanol for 60 s before adhesive application, while the control group was not. Single Bond 2 was applied in accordance with the manufacturer’s instructions to the wet demineralized dentin surface. Composite build-ups for all the groups were constructed with the light-cured 3M Z250 by using a PTFE (polytetrafluoroethylene) split mold (*r* = 1 mm). The split mold was gently and carefully removed after polymerization.

From each group, 16 specimens were stored in distilled water at 37 °C for 24 h before the shear bond test using a universal test machine (EZ-L, Shimadzu, Kyoto, Japan) in line with the standard ISO/TS 11405-2003. The fractured interfaces of all the de-bonded specimens were collected and examined with a stereomicroscope (SMZ 1500, Nikon, Tokyo, Japan) at 10× magnification to determine the de-bonding modes as follows. Adhesive failure (AF): failure occurred at the dentin/adhesive interface or the adhesive/resin interface; cohesive failure (CF): failure occurred at the inside of the resin or dentin; mixed failure (MF): both adhesive failure and cohesive failure.

The remaining four specimens of each group were cut flat perpendicular to the bond line with a low speed saw (Isomet 1000, Buehler Ltd., Lake Bluff, IL, USA) to obtain 1.0–1.5-mm-thick disks for observation of the hybrid layer. A scanning electron microscopy (S-4800, Hitachi, Tokyo, Japan) was used to evaluated the morphology of the hybrid layer after samples were sputter-coated with gold palladium for 60 s at 10 Ma (SCD050, Bal-Tec Co., Balzers, Liechtenstein).

#### 4.2.3. Cross-Link Degree Evaluation and DSC Analysis

For this part of the study, 70 extracted sound molars were used. After removal of the tooth enamel, pulpal soft tissue, and root, all dentin pieces (only coronal dentin) were obtained and dehydrated in acetone for 5 min, then triturated to a fine powder with a stainless steel mixer mill (Moderl MM400, Retsch, Newtown, PA, USA) in a liquid nitrogen atmosphere for 30 min before two-sieve screening (mesh sizes: 15 and 30 μm). The mineralized dentin was demineralized completely in 10 wt % phosphoric acid for 24 h then rinsed in MilliQ water by repeated centrifugation for 20 min at 3000 rpm in a 4 °C atmosphere and lyophilized.

The demineralized dentin powder was equally divided to seven groups by weight according to the surface treatment: no pretreatment (control), 80% ethanol treatment, and five concentrations of EDC ethanol solutions. Group 1: no treatment, used as the control; Group 2: treated with 80% ethanol for 1 min, then centrifuged in MilliQ water for 10 min at 1500 rpm, three times. The supernatant was discarded in every repeated procedure; Groups 3–7: treated with different concentrations of EDC ethanol solution for 1 min, then centrifuged in acetone for 10 min at 1500 rpm, three times. This step was to clear out the residual EDC molecules. Then, the precipitate was re-suspended in MilliQ water by centrifugation for 10 min at 1500 rpm, three times.

Thereafter, all the precipitate left in the final centrifugation was dried in an anhydrous atmosphere with SiO_2_ for at least 24 h before the cross-link degree evaluation and differential scanning calorimetry test.

Cross-link degree evaluation: the cross-linking degree was determined through the reaction of ninhydrin (2,2-dihydroxy-1,3-indanedione) with the primary amine groups of collagen according to the absorption spectroscopy method [46]. Specimens (*n* = 6) were heated with ninhydrin solution (Sino-pharm Chemical Reagent Co., Ltd., Beijing, China) for 20 min, and then the optical absorbance at 570 nm was measured with a microplate reader (Enspire, PerkinElmer, Waltham, MA, USA) at 570 nm, using glycine at various known concentrations as the standard. The amount of free amino groups in each group, after heating with ninhydrin, is proportional to the optical absorbance of the solution. Then the cross linking degree was determined.

DSC analysis: the remaining treated and untreated demineralized dentin powder from each group was subdivided into 10 specimens (10 mg each), and were analyzed with a differential scanning calorimeter (DSC, Q20 TA Instruments, New Castle, DE, USA). All the samples in each DSC test were maintained at nearly the same temperature throughout the experiment, and the temperature program for the DSC analysis was designed such that the sample holder temperature increased linearly at a rate of 10 °C·min^−1^ from 40 to 180 °C in a nitrogen atmosphere. The thermal denaturation temperature (*T*_d_) of each sample was obtained.

### 4.3. Statistical Analysis

A one-way analysis of variance (ANOVA) and Tukey’s method was used to analyze the SBS, cross-link degree and DSC results using IBM SPSS Statistics (version 20.0, IBM SPSS, Chicago, IL, USA). Furthermore, the frequency of the failure modes was analyzed using a chi-square test and Fisher’s exact test. A significance level of 0.05 was set for all statistical comparisons.

## Figures and Tables

**Table 1:** Shear bond strength (MPa) of resin-dentin with different surface treatments (mean ± standard deviation).

Surface Treatments	Shear Bond Strength (MPa)
Control	29.51 ± 5.28 ^A^
80% ethanol	29.77 ± 4.69 ^A^
0.01 M EDC	29.42 ± 3.57 ^A^
0.1 M EDC	28.64 ± 5.36 ^A^
0.3 M EDC	30.33 ± 4.37 ^A^
0.5 M EDC	29.38 ± 4.71 ^A^
1.0 M EDC	21.00 ± 3.18 ^B^

Identical superscripted upper case letters indicate no significant differences between groups (*P* > 0.05).

**Table 2:** Cross-link degree and denaturation temperature (*T*_d_/°C) of demineralized dentin powder with and without EDC treatment (means ± standard deviation).

Groups	Treatment	Cross-Link Degree (%)	Temperature Degradation (Td/°C)
G1	Control	20.62 ± 1.84 ^A^	108.81 ± 2.80 ^A^
G2	80% ethanol	21.10 ± 2.53 ^A^	108.68 ± 2.84 ^A^
G3	0.01 M EDC	25.26 ± 1.71 ^B^	113.89 ± 2.89 ^B^
G4	0.1 M EDC	26.67 ± 1.57 ^B^	115.55 ± 3.36 ^B^
G5	0.3 M EDC	37.91 ± 2.15 ^C^	120.55 ± 3.73 ^C^
G6	0.5 M EDC	39.06 ± 2.25 ^C^	120.14 ± 3.89 ^C^
G7	1.0 M EDC	37.37 ± 2.47 ^C^	115.74 ± 1.85 ^B^

Significant differences are indicated by different superscripted upper case letters in the same column (*P* < 0.05).
